# 25-vitamin D, 1,25-vitamin D, parathyroid hormone, fibroblast growth factor-23 and cognitive function in men with advanced CKD: a veteran population 

**DOI:** 10.5414/CN108365

**Published:** 2014-09-10

**Authors:** Anna J. Jovanovich, Michel Chonchol, Christopher B. Brady, James D. Kaufman, Jessica Kendrick, Alfred K. Cheung, Kristen L. Jablonski

**Affiliations:** 1 Division of Renal Diseases and Hypertension, University of Colorado Denver Anschutz Medical Campus, Aurora, CO,; 2 Research and Development Service, Veterans Affairs Boston Healthcare System, Boston, MA,; 3 Division of Nephrology and Hypertension, University of Utah, Salt Lake City, UT, and; 4 Renal Section, Medical Service, Veterans Affairs Salt Lake City Healthcare System, Salt Lake City, UT, USA

**Keywords:** chronic hemodialysis, chronic kidney disease, cognition, mineral metabolism

## Abstract

**** **Abstract. **Cognitive impairment is common in advanced chronic kidney disease (CKD), but little is known about its relation with abnormalities in mineral metabolism. Methods: The longitudinal association between plasma 25-hydroxyvitamin D (25(OH)D), 1,25-dihydroxyvitamin D (1,25(OH)_2_D), intact parathyroid hormone (iPTH), and fibroblast growth factor-23 (FGF-23) levels and cognitive function was assessed in 605 patients (67 ± 12 years) with advanced CKD not requiring dialysis (n = 247) or end-stage renal disease (ESRD; n = 358) who participated in the Homocysteine Study Cognitive Function Substudy (HOSTCOG)). Cognitive function was assessed using the Telephone Interview for Cognitive Status-modified (TICSm; mean follow-up 3.1 years) and associated with baseline mineral metabolite levels using linear regression analyses. Results: In unadjusted analyses, increasing log 1,25(OH)_2_D and decreasing log iPTH and FGF-23 levels were associated with worse cognitive status (p < 0.05). In fully adjusted multivariate analyses, the associations were no longer significant. Log 25(OH)D levels were not associated with cognitive function in unadjusted or adjusted analyses. Results were similar when analyzed by tertile or separately within CKD and ESRD groups. Conclusions: These results suggest that mineral metabolism dysregulation does not mediate the impairment in cognitive function common in advanced CKD.

**Appendix is available for free at: http://www.clinnephrol.com; Vol. 82, November 2014**

## Introduction 

Progression of chronic kidney disease (CKD) is independently associated with a decline in cognitive function [1, 2], which can result in reduced quality of life [3], increased hospitalization [4], and increased mortality [5]. Sub-clinical cerebrovascular disease is also common in advanced CKD [6, 7] and is a major risk factor for cognitive impairment [8, 9], in part by reducing cerebral blood flow [3, 10]. Recent evidence suggests that vascular disease in CKD may be systemic, and cognitive impairment may be a reflection of parallel disease processes occurring in both the kidney and the brain [11, 12]. 

With declining kidney function, there is also a progressive alteration in markers of mineral metabolism, including changes in circulating levels of vitamin D metabolites, intact parathyroid hormone (iPTH), and fibroblast growth factor-23 (FGF-23) [13]. These alterations in mineral metabolism in CKD may mediate the development of cognitive dysfunction by promoting cerebrovascular dysfunction [14, 15, 16]. However, to date, the association between a comprehensive panel of markers of mineral metabolism and cognitive function in patients with CKD has not been examined. 

To this end, we assessed the longitudinal association between plasma levels of 25(OH)D, 1,25(OH)_2_D, iPTH, and FGF-23 and cognitive function in 605 patients with advanced CKD not requiring dialysis (n = 247) and end-stage renal disease (ESRD; n = 358) who participated in the multi-center randomized controlled Department of Veterans Affairs Cooperative Study Program Homocysteine Study Cognitive Function Substudy (HOSTCOG). As there was no change in cognitive function with the study treatment of a daily high dose B-vitamin capsule [17], we were able to combine the active and placebo groups to determine whether baseline levels of markers of mineral metabolism were predictive of cognitive function as assessed at a mean follow-up of 3.1 years. 

## Methods 

### Study design (Figure 1) 

Additional details of the study design and other methods are provided in the online only data supplement (available at http://www.clinnephrol.com). This was a post-hoc analysis on the longitudinal association between mineral metabolites and cognitive function in the VA HOSTCOG study, and the statistical approach was determined *a priori*. Details of the parent VA HOST study [18, 19], conducted between 2001 and 2006 at 36 VA medical centers, and the substudy HOSTCOG [17] have been reported previously (clinicaltrials.gov identifier: NCT00032435). A battery of tests (described below) was used to assess cognitive function during the enrollment period and 1 year later. There was no effect of treatment with the high-dose B-vitamin capsule on cognitive outcomes, thus only the initial cognitive function testing (as assessed during the follow-up period of the HOST parent study (mean follow-up 3.1 ± 1.3 years)) was included in the current statistical analyses. All participants provided written informed consent. 

### Cognitive measures 

The details of the cognitive test battery in HOSTCOG have been described previously [17]. Briefly, a 20-minute battery of three cognitive tests was administered over the telephone, including the telephone interview of cognitive status-modified (TICSm), a well-validated [20, 21, 22] exam that assesses orientation, concentration, memory, responsive naming, comprehension, calculation, reasoning, and judgment. The details of the cognitive and memory composite are provided in the supplement (available at http://www.clinnephrol.com). 

## Mineral metabolism assessment 

25(OH)D, 1,25(OH)_2_D, iPTH, and FGF-23 were assayed in plasma samples stored from the 3-month post-randomization blood draw. C-terminal FGF-23 concentrations were measured using a two-site second-generation ELISA kit (Immutopics, San Clemente, CA, USA) [23]. Both intact and c-terminal FGF-23 measurements are susceptible to proteolytic degradation after 2 hours, however, this effect appears immediately for intact FGF23 [24]. Additionally, c-terminal FGF23 measurements were recently found to have less intra-individual variation [25], suggesting that it may be a more precise measurement. Plasma 25(OH)D concentrations were measured by a commercial competitive chemiluminescent immunoassay (DiaSorin, Stillwater, MN, USA) on a Liaison analyzer, and 1,25(OH)_2_D was measured by a commercial competitive radioimmunoassay (DiaSorin). Plasma iPTH measurements were performed using an electrochemiluminescent immunoassay. 

## Statistical analyses 

The associations of 25(OH)D, 1,25(OH)_2_D, iPTH, and FGF-23 with TICSm, the global cognitive z-score composite, and memory z-score composite were assessed with linear regression models. All analyses evaluated tertiles of each marker of mineral metabolism, with the highest tertile serving as the reference group. In addition, we examined each metabolite as a continuous predictor variable after log_10_ transformation. Two-tailed values of p < 0.05 were considered statistically significant. All statistical analyses were performed with SAS software, version 9.13 (SAS Institute, Cary, NC, USA). 

## Results 

### Demographic characteristics and markers of mineral metabolism 

The demographic characteristics of advanced CKD, ESRD, and all participants are shown in Table 1. Serum phosphorus, plasma iPTH and FGF-23 levels were all significantly higher in ESRD compared to CKD patients, and plasma 25(OH)D and 1,25(OH)_2_D levels were lower (Table 1). 

### Measures of cognitive function 

As reported previously in HOSTCOG [17], cognitive function impairment, quantified as a TICSm score of ≤ 27 (maximum score is 50), was detected in ~ 19% of patients, regardless of treatment (high-dose vitamin B or placebo) or kidney disease status (CKD or ESRD). Table 1 shows cognitive function of the participants included in this analysis by group (CKD or ESRD), assessed as the total TICS score, global cognitive z-score composite, and memory z-score composite. Each variable did not differ between the CKD and ESRD groups. 

### Relation between mineral metabolites and cognitive function 

Increasing plasma log_10_ 25(OH)D levels were not associated with performance on the TICSm in unadjusted analyses or after multivariate adjustment for age and race (model 1), model 1 plus BMI, smoking status, years of education, homocysteine level, treatment group, diabetes, hypertension, cardiovascular diseases, systolic blood pressure, and diastolic blood pressure (model 2), or model 2 plus corrected serum calcium, serum phosphorus, 25(OH)D, iPTH, and FGF-23 (model 3) (Table 2). Results were similar when 25(OH)D was analyzed by tertiles (Table 2). 

Higher plasma log_10_ 1,25(OH)_2_D levels were associated with worse cognitive function in unadjusted analyses, but not after multivariate adjustment in all three models (Table 2). Increasing log_10_ PTH levels were associated with greater cognitive function in unadjusted analyses only, with no association after multivariate adjustment in all models (Table 2). Similarly, higher levels of FGF-23 were associated with greater performance on the TICSm in unadjusted analysis only, with no association after multivariate adjustment (Table 2). 

Results were similar when CKD and ESRD groups were analyzed separately, with no association between the markers of mineral metabolism and TICSm score in any of the adjusted models (Supplemental Table 1 and Supplemental Table 2 available at http://www.clinnephrol.com); p for interaction > 0.60 for all). Associations were also non-significant in all adjusted analyses when the cognitive or memory z-score composite were considered individually as the outcome of interest. Finally, the associations of the markers of mineral metabolism with change in cognitive function after 1 year of treatment were also non-significant in all adjusted analyses. 

## Discussion 

In a large sample of advanced CKD and chronic dialysis patients who participated in the HOSTCOG study, there was no independent association between plasma 25(OH)D, 1,25(OH)_2_D, iPTH, or FGF-23 and TICSm score, a measure of cognitive function. This finding persisted when advanced CKD and chronic hemodialysis (HD) groups were analyzed separately, and when the cognitive and memory z-score composites were considered as dependent variables. Thus, in the first study to evaluate the association between multiple markers of mineral metabolism and cognitive function and CKD, we found no evidence to support an independent association. 

It has been proposed that higher circulating levels of 25(OH)D may preserve cognitive function via its vasculoprotective and neuroprotective properties [26]. 25(OH)D deficiency is associated with vascular endothelial dysfunction [16, 27], which is mediated in part by increased vascular inflammation [27], and vascular endothelial dysfunction is associated with impaired cognitive function [28]. In addition, the vitamin D receptor is present in the brain, and vitamin D may be neuroprotective through mechanisms including antioxidant activity, calcium regulation, immunomodulation, enhanced nerve conduction, and detoxification [26]. 25(OH)D levels have been independently associated with current as well as longitudinal decline in cognitive function in the general population in several [29, 30, 31], but not all [32] studies. In a recent study of 225 chronic HD patients, Shaffi et al. [33] found that a higher 25(OH)D serum level was independently associated with better performance on executive function tests, but not memory tests. In contrast, we found no relation in a larger sample size of advanced CKD and chronic HD patients between 25(OH)D levels and cognitive performance. Of note, our study differed from Shaffi et al. in that our design was longitudinal, included patients with advanced CKD not on chronic HD, multivariate models included adjustment for other mineral metabolites, and our sample was primarily men. The association between plasma 1,25(OH)_2_D levels and cognitive function has not been previously evaluated, either in patients with CKD or in general population. We also found no significant independent association between plasma 1,25(OH)_2_D levels and measures of cognitive function. 

It has been suggested that increased levels of parathyroid hormone may also be associated with cognitive dysfunction, although the mechanism is unknown [34, 35]. In patients free from CKD who have primary hyperparathyroidism, parathyroidectomy is associated with improved cognitive function [36, 37]. In a small study of patients with secondary hyperparathyroidism (due to calcium deficit) without impaired renal function, elevated iPTH levels were also associated with impaired cognitive function [38]. Furthermore, an improvement in cognitive function with nocturnal daily HD may partially be explained by a decrease in iPTH levels [39]. However, we found no independent association between iPTH levels and cognitive function in patients with advanced CKD or ESRD. 

The relation between FGF-23 and cognitive function is largely unknown, both in patients with kidney disease or in any population. A recent cross-sectional analysis in chronic hemodialysis patients found an independent association between FGF-23 levels and a worse composite memory score [40]. It is possible that FGF-23 may mediate cognitive function via the promotion of vascular dysfunction [11, 14, 41]. However, our results do not support an independent association between FGF-23 levels and cognitive function in the HOSTCOG cohort. 

Collectively, the lack of association between 25(OH)D, 1,25(OH)_2_D, iPTH, and FGF-23 levels suggests that mechanisms other than alterations in mineral metabolisms contribute to the decline in cognitive function with advancing CKD. This may include traditional factors known to influence cognitive function such as age, hypertension, diabetes, hypercholesterolemia, and cigarette smoking [3, 8], as well as non-traditional risk factors may also mediate cognitive dysfunction, including hemostatic abnormalities, hypercoaguable states, sleep disturbances, anemia, and depression [3, 8]. In addition, it is also possible that vascular dysfunction, perhaps mediated by oxidative stress, inflammation and other uremic toxins, contributes to the decline in cognitive function independent of alterations in mineral metabolism. 

There are several important limitations of the present study, including carryover of the limitations in the original HOSTCOG study to this post-hoc analysis [17]. Importantly, these data are observational in nature, thus do not provide evidence of causation. In addition, due to the design of HOSTCOG, cognitive function measurements were not available at the same time point as the assessment of mineral metabolism, thus there is a time lag between these measurements (mean follow-up 3.1 ± 1.3 years), and no data available regarding changes in markers of mineral metabolism over time. Also, cognitive function assessment by telephone may not be as sensitive as other in-person assessment tools, particularly in an ill population, as was included in HOST. Although the circulating levels of mineral metabolism markers differed in CKD and ESRD, cognitive function testing was identical across the spectrum of kidney disease status, and results were similar when CKD and ESRD groups were analyzed separately. 

Also, information on the use of nutritional vitamin D supplements or active vitamin D analogues was unavailable from HOST. However, given the time period in which HOST was performed, vitamin D analogues and vitamin D supplementation were not prevalent in the non-dialysis patients, thus limiting potential confounding by these variables. Similarly, medication reconciliation was not performed during HOST, and changes in vitamin D supplementation across time were not measured, which is a major limitation that carries over to the present analysis. Last, the cohort is nearly entirely men (a Veteran population), thus it is unknown if the association between mineral metabolism and cognitive function may differ in women. Important strengths of the study include a relatively large sample size and novel insight into the association of four markers of mineral metabolism with cognitive function in patients with advanced CKD and ESRD. 

In conclusion, in the HOSTCOG cohort, plasma 25(OH)D, 1,25(OH)_2_D, iPTH, and FGF-23 levels did not independently predict TICSm score, a measure of cognitive function. Thus, while dysregulation of mineral metabolism is certainly an important contributor to other adverse outcomes, these results suggest that this dysregulation does not mediate the impairment in cognitive function that is common in advanced CKD and ESRD. 

## Acknowledgments 

This work was supported by the American Heart Association and the National Institutes of Health (12POST11920023; and 1R01DK081473-01). 

## Conflict of interest 

None. 

**Figure 1.**
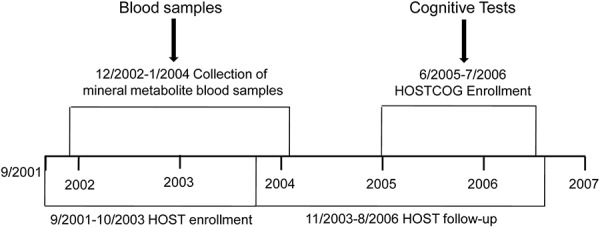
Department of Veterans Affairs Cooperative Study Program Homocysteine Study (HOST), HOST Cognitive Function Substudy (HOSTCOG), and mineral metabolite sample timeline.

**Table 1.** Demographic characteristics, markers of mineral metabolism, and measures of cognitive function of the whole population and by kidney disease status.

	Total (n = 605)	CKD (n = 247)	ESRD (n = 358)	p-value
Age (y)	67 ± 12	69 ± 12	66 ± 11	0.001
Male sex	595 (98%)	243 (98%)	352 (98%)	0.9
Race				0.0002
White	304 (50%)	67 (27%)	149 (42%)	
Black	216 (36%)	148 (60%)	156 (44%)	
Other	85 (14%)	32 (13%)	53 (15%)	
Education (y)	13 ± 3	13 ± 3	13 ± 3	0.5
Cause of CKD				0.01
Diabetes	233 (39%)	73 (30%)	160 (45%)	
GN	38 (6%)	17 (7%)	21 (6%)	
HTN	211 (35%)	96 (39%)	115 (32%)	
OU	13 (2%)	4 (2%)	9 (3%)	
PKD	26 (4%)	14 (6%)	12 (3%)	
Other	84 (14%)	43 (18%)	41 (11%)	
Diabetes	299 (49%)	98 (40%)	201 (56%)	< 0.0001
Hypertension	585 (97%)	240 (97%)	345 (96%)	0.7
CVD	299 (49%)	116 (47%)	183 (51%)	0.3
Smoking				
Current	106 (18%)	40 (16%)	66 (18%)	0.9
Former	334 (55%)	137 (56%)	197 (55%)	0.5
BMI (kg/m^2^)	28 ± 5	27 ± 5	29 ± 5	0.003
SBP (mmHg)	142 ± 23	141 ± 23	142 ± 23	0.4
DBP (mmHg)	75 ± 13	74 ± 13	76 ± 13	0.08
Homocysteine (mmol/L)	24 ± 7	23 ± 7	24 ± 7	0.10
Calcium* (mg/dL)	8.98 ± 0.85	9.00 ± 0.91	8.96 ± 0.80	0.5
Phosphorus (mg/dL)	4.74 ± 1.57	4.27 ± 1.37	5.07 ± 1.62	< 0.0001
25(OH)D (ng/mL)	18 [12 – 25]	19 [13 – 28]	17 [11 – 24]	0.001
1,25(OH)_2_D (pg/mL)	16 [11 – 23]	19 [13 – 27]	14 [10 – 20]	< 0.0001
iPTH (pg/mL)	154 [93 – 280]	111 [75 – 186]	185 [114 – 345]	< 0.0001
FGF-23 (RU/mL)	657 [254 – 2,959]	288 [181 – 1,003]	1,135 [390 – 4,238]	0.0001
Total TICSm score	32.04 ± 5.12	32.01 ± 5.30	32.06 ± 5.00	0.9
Global cognitive z-score composite	0.01 ± 0.71	0.03 ± 0.68	0.00 ± 0.72	0.7
Memory z-score composite	0.01 ± 0.82	–0.03 ± 0.86	0.03 ± 0.79	0.3

Data are expressed as number (%), mean ± SD, or median [interquartile range]. *Corrected for serum albumin concentration. GN = glomerulonephritis; HTN = hypertension; OU = obstructive nephropathy; PKD = polycystic kidney disease; CVD = cardiovascular disease; BMI = body mass index; SBP = systolic blood pressure; DBP = diastolic blood pressure; 25(OH)D = 25-hydroxyvitamin D; 1,25(OH)_2_D = 1,25-dihydroxvitamin D; iPTH = intact parathyroid hormone; FGF-23 = fibroblast growth factor-23; TICSm = Telephone Interview of Cognitive Status-modified.

**Table 2.** Associations (β-estimates; 95% CI) of markers of mineral metabolism with Telephone Interview of Cognitive Status-modified (TICSm) score.

	Tertile 1	Tertile 2	Tertile 3	Continuous
25(OH)D	< 13 ng/mL	13 – 22 ng/mL	22 ng/mL	Log_10_ 25(OH)D
Unadjusted	0.39 [– 0.63, 1.41]	–0.31 [– 1.31, 0.69]	Ref	–0.79 [–2.48, 0.95]
Model 1	0.14 [– 0.84, 1.12]	–0.02 [– 0.92, 0.88]	Ref	–0.22 [–1.93, 1.49]
Model 2	0.01 [– 0.95, 0.97]	0.10 [– 0.78, 0.98]	Ref	0.06 [–1.61, 1.73]
Model 3	0.16 [– 0.88, 1.2]	0.18 [– 0.74, 1.10]	Ref	–0.23 [–2.09, 1.63]
1,25(OH)_2_D	< 11 pg/mL	11 – 19 pg/mL	> 20 pg/mL	log_10_ 1,25(OH_2_D
Unadjusted	1.26 [0.26, 2.26]*	1.60 [0.62, 2.58]*	Ref	–1.97 [–3.68, –0.26]*
Model 1	0.07 [– 0.87, 1.01]	0.92 [0.04, 1.80]*	Ref	0.09 [–1.52, 1.70]
Model 2	–0.17 [– 1.09, 0.75]	0.68 [– 0.20, 1.56]	Ref	0.50 [–1.09, 2.09]
Model 3	–0.27 [– 1.29, 0.75]	0.61 [– 0.29, 1.51]	Ref	0.75 [–1.09, 2.59]
iPTH	< 109 pg/mL	109 – 217 pg/mL	> 217 pg/mL	log_10_ iPTH
Unadjusted	–1.27 [– 2.25, –0.29]*	–1.21 [– 2.21, –0.21]*	Ref	1.14 [0.02, 2.26]*
Model 1	–0.70 [– 1.62, 0.22]	–0.74 [– 1.66, 0.18]	Ref	0.36 [– 0.68, 1.40]
Model 2	–0.71 [– 1.59, 0.17]	–0.95 [– 1.85, –0.05]*	Ref	0.38 [– 0.62, 1.38]
Model 3	–0.70 [– 1.66, 0.26]	–0.95 [– 1.89, –0.01]*	Ref	0.29 [– 0.79, 1.37]
FGF-23	< 326 Ru/mL	326 – 1574 Ru/mL	> 1574 Ru/mL	Log_10_ FGF-23
Unadjusted	–1.90 [– 2.9, –0.9]*	–0.56 [– 1.54, 0.42]	Ref	1.05 [0.48, 1.62]*
Model 1	–0.13 [– 1.13, 0.87]	0.17 [– 0.75, 1.09]	Ref	0.02 [– 0.55, 0.59]
Model 2	–0.28 [– 1.26, 0.70]	–0.12 [– 1.02, 0.78]	Ref	0.13 [– 0.44, 0.70]
Model 3	–0.46 [– 5.78, 0.72]	–0.25 [– 1.25, 0.75]	Ref	0.23 [– 0.78, 0.94]

Model 1: age, race. Model 2: model 1, body-mass index, smoking status, years of education, homocysteine level, treatment group, diabetes, hypertension, cardiovascular disease, systolic blood pressure, diastolic blood pressure. Model 3: Model 2, calcium, phosphorus and other markers of mineral metabolism (25-hydroxyvitamin D, 1,25-dihydroxyvitamin D, parathyroid hormone, and/or fibroblast growth factor-23, depending on variable of interest). *p < 0.05.
